# Integrating high-throughput phenomics and GWAS unravels the HaCBF4-*HaHAK11* module to regulate salt stress tolerance in sunflower (*Helianthus annuus* L.)

**DOI:** 10.1093/hr/uhag081

**Published:** 2026-03-04

**Authors:** Weijun Guo, Jiawei Qiu, Youling Zeng, Xinxin Li, Shurui Dong, Qinyang Li, Yushan Liu, Maohong Cai, Zhonghua Lei, Tao Chen

**Affiliations:** College of Life and Environmental Science, Hangzhou Normal University, Hangzhou City, Zhejiang Province 311121, China; College of Life Sciences, South China Agricultural University, Guangzhou City, Guangdong Province 510642, China; College of Life and Environmental Science, Hangzhou Normal University, Hangzhou City, Zhejiang Province 311121, China; College of Life Science and Technology, Xinjiang University, Urumqi, Xinjiang Uygur Autonomous Region 830046, China; College of Life and Environmental Science, Hangzhou Normal University, Hangzhou City, Zhejiang Province 311121, China; College of Life and Environmental Science, Hangzhou Normal University, Hangzhou City, Zhejiang Province 311121, China; College of Life and Environmental Science, Hangzhou Normal University, Hangzhou City, Zhejiang Province 311121, China; College of Life and Environmental Science, Hangzhou Normal University, Hangzhou City, Zhejiang Province 311121, China; College of Life and Environmental Science, Hangzhou Normal University, Hangzhou City, Zhejiang Province 311121, China; Institute of Economic Crops, Xinjiang Academy of Agricultural Sciences, Urumqi, Xinjiang Uygur Autonomous Region 830046, China; College of Life and Environmental Science, Hangzhou Normal University, Hangzhou City, Zhejiang Province 311121, China

## Abstract

Sunflower (*Helianthus annuus* L.) is one of the pioneer crops with extremely strong adaptability to adverse stresses, and its stress (such as high salinity) tolerance improvement will contribute to the utilization of abundant marginal land and promote sustainable development. However, the genetic determinants underlying response to salt stress are not fully understood. Here, we perform a genome-wide association study (GWAS) using 31 traits from a high-throughput platform in 342 oilseed sunflower accessions at the germination stage under salt stress conditions. We identify 359 significantly associated SNPs and 63 InDels corresponding to 424 and 83 candidate genes, respectively. One candidate gene, *C-Repeat Binding Factor 4* (*CBF4*)—a member of the AP2/EREBP family transcription factor—directly binds to dehydration-responsive element in the promoter region of its downstream target gene, *High-Affinity K*^*+*^  *transporter 11* (*HAK11*), thereby activating its expression. This regulatory mechanism contributes to enhanced salt tolerance in sunflower by modulating established salt-responsive genetic pathways. Collectively, our findings provide new insights into salt tolerance mechanisms and offer valuable genetic resources for breeding salt-tolerant sunflower cultivars.

## Introduction

With the continuous growth of the global population, food security has become a major concern, a challenge further exacerbated by the potential impact of climate change on crop productivity. Soil salinity, extreme temperatures, and drought are key environmental stresses affecting plants; among these, soil salinity is a critical factor threatening global agricultural productivity and ecological stability [1]. Specifically, saline soils are characterized by the accumulation of excessive soluble salts, which serve as the primary environmental factor limiting seed germination, crop growth, and yield [2]. Globally, over 900 million hectares of land are affected by salinity, approximately one-third being irrigated agricultural land. Furthermore, driven by climate change and anthropogenic activities, this issue is intensifying, causing estimated annual economic losses of 27.3 billion US dollars [3, 4]. Therefore, the utilization of saline land is of great significance for ensuring food security.

Sunflower (*Helianthus annuus* L.) is a premier oilseed crop, which is native to North America and currently cultivated extensively worldwide, and has been recognized as a nutraceutical and functional food due to its beneficial health effects [5]. The crop’s robust adaptability to diverse soil and climatic conditions has facilitated its widespread global cultivation [6]. Driven by the continuous growth of the global population, the demand for sunflower seeds, oil, and by-products has risen significantly. Consequently, there is an urgent need to expand sunflower production. However, soil salinity represents a major abiotic stress that affects plant growth and development, thereby compromising crop yield and quality [1]. Although sunflower is generally considered a moderately salt-tolerant (ST) crop [7], for instance, the germination rate (GR) of the parental line ZSADT was not affected under 200 mM NaCl solution treatment [8, 9]; further improvement is necessary. Therefore, identifying key genetic determinants and breeding salt-tolerance varieties are essential strategies to enhance the utilization of salinity land and boost the yield to meet global demands.

When combined with high-throughput phenotyping, genome-wide association study (GWAS) has proven to be a powerful approach for dissecting the genetic architecture of complex traits and abiotic stress responses in plants [8–14]. In sunflowers, several GWAS have investigated the genetic basis of both biotic and abiotic stresses [15–18]. Although candidate genes associated with these traits have been identified, their underlying molecular mechanisms remain poorly understood, which hinders their application in molecular breeding programs. Furthermore, the limited number and relatively low genome coverage of genetic variations detected through array-based genotyping or genotyping-by-sequencing in previous studies have constrained the accuracy and efficiency of candidate gene discovery. Therefore, it is essential to utilize whole-genome sequencing (WGS) to construct a comprehensive and high-resolution variation dataset to support more robust downstream association analyses.

In response to salt stress, plants have developed various molecular mechanisms, including ion homeostasis regulation, ion compartmentalization, and osmoprotectant biosynthesis [2, 19]. A large number of proteins involved in these response mechanisms have been identified, and their molecular regulation has been extensively characterized [1, 19]. Specifically, transcription factor (TF) families such as AP2/ERF (APETALA2/Ethylene Responsive Factor), NAC, bZIP (basic region/leucine Zipper), MYB (Myeloblastosis), and WRKY regulated plant salt tolerance by modulating the expression of known salt-responsive genes [20]. Among these, the AP2/ERF family, one of the largest groups of plant-specific TF, plays critical roles in the regulation of metabolism, growth, and development programs, as well as responses to environmental stress [21, 22]. Members of this family contain a conserved AP2/ERF DNA binding domain, allowing them to regulate gene expression by binding to *cis*-elements in the promoters of target genes. The AP2/ERF superfamily is composed of four subfamilies: AP2 (APETALA2), DREB (dehydration-responsive element-binding proteins), ERF (ethylene-responsive factors), and RAV (related to ABI3/VP1) [23]. Although previous studies have extensively reported the role of AP2/ERF family members in abiotic stress response, the specific functional mechanisms of these TFs in sunflowers under salt stress remain unclear.

Crop growing in the salinized farmlands uptakes a large amount of sodium ions (Na^+^), which are the most abundant soluble cations in saline soils. These ions are transported to aboveground tissues, where they disrupt various physiological processes, such as photosynthesis, growth, and nutrient uptake [24–26]. The exclusion of Na^+^ from shoot tissue is a critical physiological process controlled by Na^+^ preferential transporters and their regulators [24, 25, 27], enabling crops to tolerate the salt stress [2, 19]. Previous studies have reported that Na^+^ preferential transporters encoded by High-Affinity K^+^ transporter (HAK) family genes, such as *OsHAK12* and *ZmHAK4*, modulate salt tolerance in crops by promoting Na^+^ exclusion from shoots [28–30]. These findings provide novel insights into the mechanism of shoot Na^+^ exclusion and offer promising genetic resources for the molecular breeding of ST crops. Therefore, understanding the transcriptional and post-transcriptional mechanisms of HAK family Na^+^ transporters regulation will facilitate their application in breeding ST crops.

In this study, we perform a large-scale GWAS using high-throughput phenotyping of 342 oilseed sunflower accessions under salt stress conditions. We construct a high-density variation map using WGS and identify the key candidate gene *HaCBF4*, an AP2/EREBP family TF, which directly binds to the promoter of the target gene, *high-affinity K*^*+*^  *transporter 11* (*HAK11*), and activates its expression to positively regulate salt tolerance in sunflower. Our findings provide new insights into the transcriptional regulation of sunflowers under salt stress and contribute to the molecular breeding and genetic improvement of salt tolerance in sunflowers.

## Results

### Phenotypic variation of traits in sunflower under salt stress

**Figure 1:**
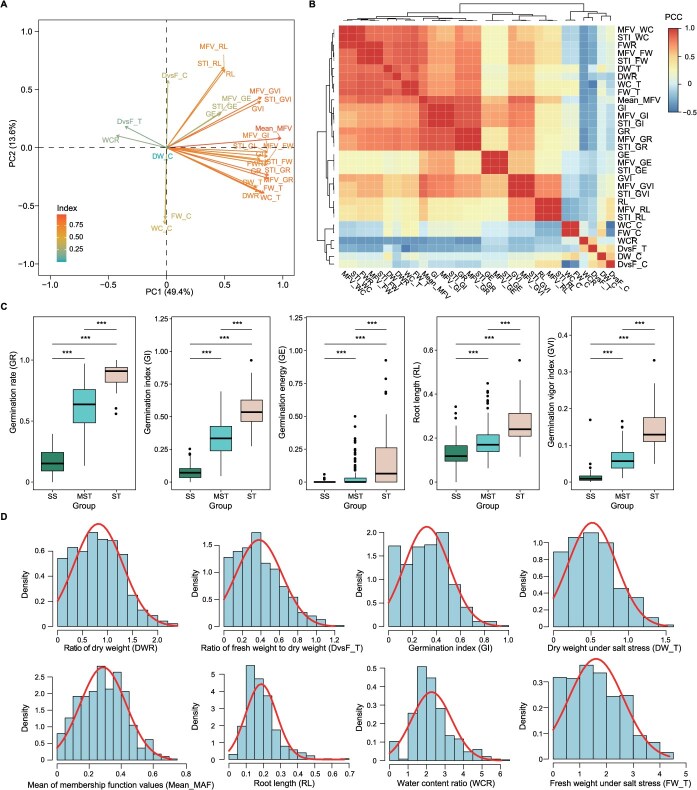
High-throughput phenotyping of 342 populations under salt stress at the germination stage in sunflower. (A) PCA of 31 traits acquired from a high-throughput platform at the germination stage under salt stress. (B) The heatmap shows the correlation analysis among 31 traits. The detailed description of traits was also listed in the methods and Table S1. PCC represents the Pearson correlation coefficient. (C) Comparison of five representative traits in three different salt tolerance populations. SS, MST, and ST represent salt-sensitive, moderate salt-tolerant, and salt-tolerant sunflowers. (D) Distribution of traits in 342 sunflower germplasms. Significance in (C) was determined using the paired Student’s *t*-test method. ^***^ represents *P <* 0.001.

To understand the phenotypic variation of sunflower in response to salt stress, we collected 342 accessions with different salt tolerance capacities, consisting of 51 ST, 218 moderate salt-tolerant (MST), and 73 salt-sensitive (SS) germplasms (Table S1), whose salt tolerance was evaluated in our previous study [8, 9]. Using a high-throughput phenotyping platform, we acquired data for a total of 31 traits, including GR, germination index (GI), germination energy (GE), root length (RL), germination vigor index (GVI), at the seed germination stage under salt stress conditions (Table S2). We performed principal component analysis (PCA) on these traits and calculated the Pearson correlation coefficients (PCCs) among phenotypes. The first two principal components (PC1 and PC2) explained 63% of the total variance (Fig. 1A and B). Key traits, including GR, GI, GE, RL, and GVI, exhibited significant differences among the three categories of sunflower accessions (Fig. 1C and Fig. S1). Frequency distribution analysis showed that the ratio of dry weight (DWR), ratio of fresh weight (FW) to dry weight (DvsF_T), GI, dry weight under salt stress (DW_T), mean of membership function values (Mean_MAF) representing the fuzzy comprehensive evaluation method (Table S2), RL, water content ratio (WCR), and FW under salt stress (FW_T) followed a normal distribution (Fig. 1D). These results indicated that the phenotype of the 342 sunflowers accessions under salt stress conditions exhibited significant natural variation, reflecting the diverse genetic backgrounds across the panel.

### GWAS for phenomics under salt stress in the oilseed population

**Figure 2:**
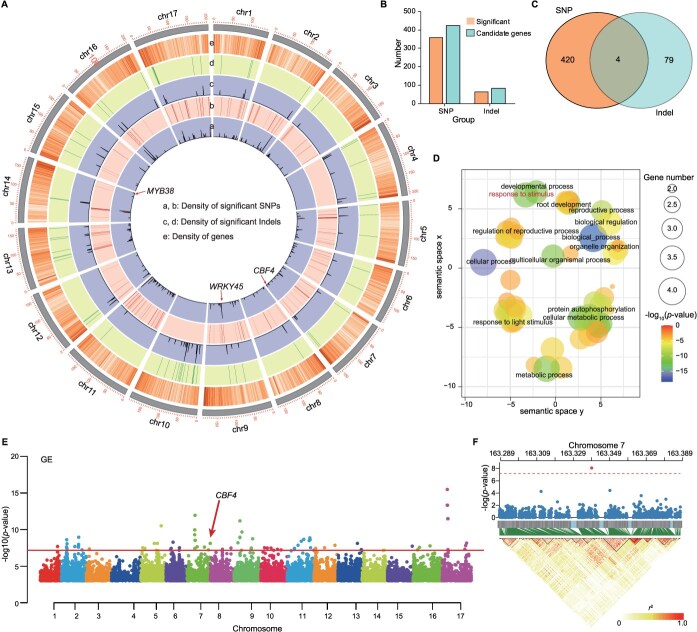
Integrated high-throughput phenotyping and GWAS to identify candidate genes in response to salt stress. (A) A Circos plot showed the chromosomal distribution of loci associated with all traits. Five rings form inside to outside show the density of significant associated SNPs (a, b), InDels (c, d), and the density of all genes (d), where a and c were line plots, b and d were heatmaps of density. Each vertical line indicated a locus. (B) The number of significant associated SNPs and InDels, and the corresponding candidate genes. (C) The venn diagram showed the number of significant associated SNPs and InDels. (D) GO enrichment results of candidate genes. The circle represents gene number in the corresponding significant enrichment terms, and the color represents significance. (E) The Manhattan plot shows the GWAS result of GE. Along the *x*-axis, the chromosome numbers were set. The red solid line represents the statistical significance cutoffs that were transformed using −log_10_(*P*-value). The red arrows indicate the candidate gene *HaCBF4*, which was selected for the next validation. The Bonferroni threshold and false detection rate (FDR) correction were used to adjust the *P*-value for reducing false positive associations. (F) Local Manhattan plot (upper) and LD statistic *r*^2^ values (lower) for the candidate gene *HaCBF4* associated with GE.

To investigate the genetic basis of salt tolerance in sunflower, we performed a GWAS to identify significantly associated variations and candidate genes (Fig. 2). All of these 342 accessions were subjected to WGS, generating a total of 16 008 Gb of data (Fig. S2A). After trimming low-quality reads, the clean data were aligned to the reference genome, and then the variations were called based on the alignment results using GATK software (Fig. S2B–E). We obtained a high-density variation map comprising 11 245 407 single-nucleotide polymorphisms (SNPs) and 1 641 341 insertions/deletions (InDels) (Fig. S2F). We analyzed linkage disequilibrium (LD) and observed rapid LD decay in both sub-populations (Fig. S2G). Using a mixed linear model (MLM) accounting for population structure and relative kinship into account, we implemented GWAS using GEMMA software with a strict significant genome wide *P*-value (6.87E−08, 0.05/effective number) determined using GEC software [31]. We identified a total of 359 trait-associated SNPs (258 unique) and 63 InDels (44 unique) corresponding to 424 and 83 genes, respectively (Fig. 2A, B; Fig. S3, and Tables S3–S5). After merging significant signals based on the LD distance (~400 kb), we defined 225 quantitative trait loci (QTL) (Table S6). A venn diagram revealed that four unique genes were associated with both SNP and InDel (Fig. 2C). Among these, candidate *HanSK16G10790* and *HanSK16G10800* were significantly associated with a SNP signal of GE and with an InDel signal with MFV_GE and STI_GE; *HanSK01G24580* was significantly associated with a SNP signal of GVI and with an InDel signal with GE; and *HanSK06G24010* was significantly associated with a SNP signal of GE and with an InDel signal with FWR (Table S3).

Gene ontology (GO) enrichment analysis showed that these candidate genes were significantly enriched in the categories ‘response to stimulus (*P* = 1.8E−04)’ and ‘metabolic process (*P* = 2.20E−05)’ (Fig. 2D; Table S7). To investigate the potential molecular mechanism underlying the salt stress response, we focused on the 121 genes significantly enriched in the ‘response to stimulus’ category. Among these, the *LOC110910181* carries an InDel (chr9_105512693: AT/A) in its downstream region and was significantly associated with the membership function value of GR (MFV_GR) (*P* = 6.26E−08) (Table S3). Its rice homolog, *OsWRKY45,* encodes a WRKY-like TF with two alleles, *OsWRKY45-1* and *OsWRKY45-2*. Notably, *OsWRKY45-2*, but not *OsWRKY45-1*, negatively regulates the salt stress response in rice [32]. Another candidate, *LOC110905118,* carries a SNP (chr14_177053528: A/G) in the upstream region, which is significantly associated with WCR (*P* = 1.05E−08) (Table S3). Its rice homolog, *OsMYB38*, encodes a MYB TF that exhibits expression induced by salt, cold, and drought stress in the drought-tolerant rice cultivar N22 [33]. Additionally, the candidate gene *LOC110869265* is homologous to *Arabidopsis thaliana C-repeat-binding factor 4* (*CBF4*), which encodes a member of DREB subfamily A-1 within the AP2/ERF TF family [34]. This gene (hereafter referred to as *HaCBF4*) carries a SNP (chr7_163340113: A/G) annotated in the promoter region and was significantly associated with GE in sunflower (*P* = 8.26E−09; Fig. 2E, F; Table S3). These findings suggest that these candidate genes may contribute to salt stress response in sunflowers.

### HaCBF4 is a positive regulator of salt stress tolerance in sunflower

**Figure 3:**
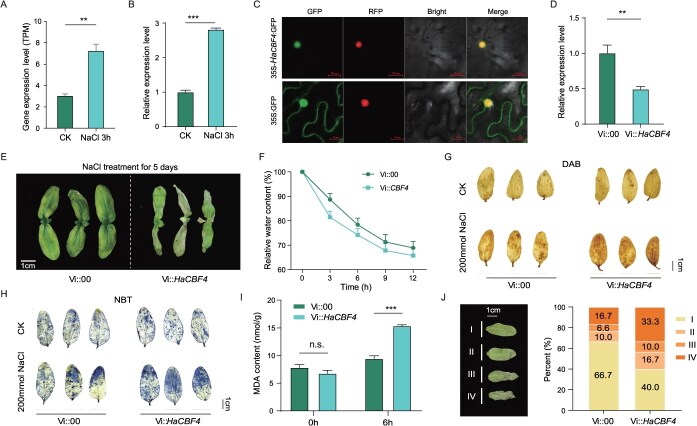
HaCBF4 positively responds to salt stress. (A) The gene expression level of *HaCBF4* at 3-week-old seedlings under normal conditions and 3 h after 200 mM NaCl solution treatment. (B) The relative gene expression level of *HaCBF4*. (C) Subcellular localization of HaCBF4 protein. (D) The relative gene expression level of *HaCBF4* in gene silencing plants (Vi:: *HaCBF4*) was significantly decreased compared to the control plants (Vi:: 00). (E) The phenotype of Vi:: *HaCBF4* and Vi:: 00 after 5 days of treatment using a 200 mM NaCl solution at 3-week-old seedlings. (F) The relative WC of Vi:: *HaCBF4* and Vi:: 00 after treatment using a 200 mM NaCl solution. (G, H) The DAB (G) and NBT (H) content of Vi:: *HaCBF4* and Vi:: 00 leaves under normal and salt stress conditions. (J) The MDA content of Vi:: *HaCBF4* and Vi:: 00 under normal conditions and after 6 h of treatment using a 200 mM NaCl solution. (I) The proportion of four damaged degrees of leaf of Vi:: *HaCBF4* and Vi:: 00 after treatment. Significance in (A), (B), (D), and (I) was determined using the paired Student’s *t*-test method. ^**^ and ^***^ represent *P* < 0.01 and *P* < 0.001, respectively. n.s. means no significant difference.

Many DREB proteins bind to the Dehydration Responsive Elements (DRE) or C-Repeat Elements (CRT) containing the A/GCCGAC core sequence located in the promoter regions of downstream genes associated with abscisic acid (ABA), drought, and cold stress responses. In *Arabidopsis*, *DREB1B* (also known as *C-Repeat Binding Factor 1* (*CBF1*)), *DREB1A* (*CBF3*), and *DREB1C* (*CBF2*) are induced by cold stress, whereas *DREB1D* (*CBF4*) is induced by drought stress [35]. In rice, *OsDREB1C*/*E*/*G* play roles in scavenging reactive oxygen species (ROS) and regulating cell death under low-temperature stress. Furthermore, these three genes act as positive regulators of heat tolerance, and *OsDREB1C*/*G* also positively regulates salt tolerance [36]. Although CBF4 plays an important role in osmotic, ABA, and drought stress responses in *Arabidopsis* [34], its function in the response to salt stress remains unknown. To investigate the potential function of HaCBF4 under salt stress, we constructed a phylogenetic tree, which revealed that HaCBF4 exhibits a close genetic relationship with homologs in dicotyledonous plant species (Fig. S4A), indicating a potentially conserved functional role across related taxa. To confirm the role of *HaCBF4* in regulating the sunflower salt stress response, we first analyzed its expression pattern using transcriptome sequencing data from multiple tissues across five representative growth stages; results showed that *HaCBF4* exhibits a spatiotemporal expression pattern (Fig. S4B and Table S8). Furthermore, *HaCBF4* expression was significantly induced under salt stress at the seedling stage based on transcriptome data, a finding confirmed by real time quantitative reverse transcription PCR (qRT-PCR) (*P* < 0.01 and 0.001) (Fig. 3A and B). Finally, we examined the subcellular localization of the HaCBF4 protein by transiently co-expressing a *35S*::*HaCBF4-GFP* fusion construct with the nuclear localization marker D53-mCherry into *Nicotiana benthamiana* leaves. The fluorescence signals mainly accumulated in the nuclei, indicating that the HaCBF4 protein is localized in the cell nucleus (Fig. 3C).

Furthermore, we generated *HaCBF4*-silenced sunflower plants using virus-induced gene silencing (VIGS), confirming the silencing efficiency by qRT-PCR (Fig. 3D). Under normal conditions, the *HaCBF4*-silenced plants showed no significant phenotypic differences compared to control plants transformed with an empty vector (Fig. S4C–H). However, after 5 days of treatment with 200 mM NaCl solution, the *HaCBF4*-silenced plants showed a more salt-sensitive phenotype than the control (Fig. 3E). Additionally, the relative water content (WC) of the *HaCBF4*-silencing plants decreased more rapidly compared with the control group (Fig. 3F). To visualize ROS accumulation, we stained the leaves from *HaCBF4*-silenced and control plants with 3,3′-diaminobenzidine (DAB) and nitroblue tetrazolium (NBT). The average staining intensity of DAB and NBT was higher in *HaCBF4*-silenced plants compared to control plants under salt stress, indicating greater ROS accumulation in the silenced plants (Fig. 3G–H). We also measured malondialdehyde (MDA) content. While no significant difference was observed under normal conditions, MDA levels were significantly higher in *HaCBF4*-silenced plants compared to the control group under salt stress (*P* < 0.001) (Fig. 3I). Finally, we classified damaged leaves into four categories (group I–IV) based on the degree of curling after salt stress. The percentage of category IV (severe curling) increased from 16.7% in the control group to 33.30% in *HaCBF4*-silenced plants, confirming that these plants are more sensitive to salt stress than the control (Fig. 3J). Collectively, these results suggest that *HaCBF4* positively regulates salt stress response in sunflower.

### Integrated transcriptome and bioinformatics analysis identify the target gene

**Figure 4:**
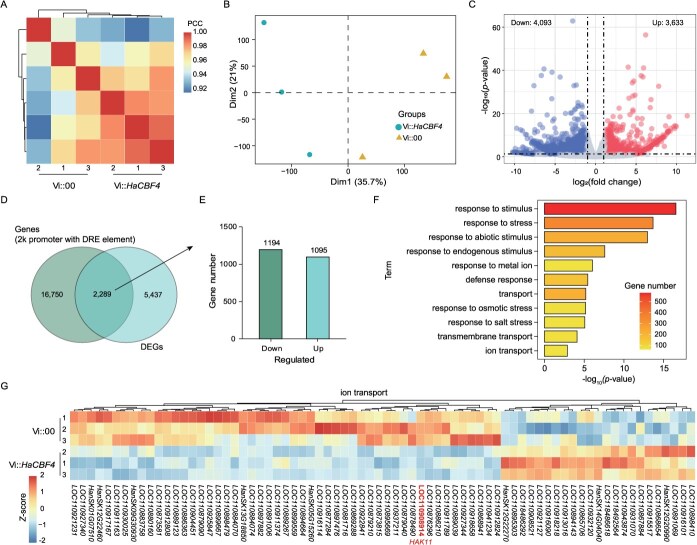
Comparison analysis of Vi:: *HaCBF4* transcriptome under salt stress conditions. (A) PCCs of three biological replicates of control (Vi:: 00) and gene silencing sample (Vi:: *HaCBF4*). (B) PCA of transcriptomes. (C) Volcano plot showed the number of DEGs that were divided into up- and down-regulated categories. (D) Venn plot of DEGs and genes that 2 kb promoter contained DRE motif (A/GCCGAC). (E) Overlapped 2289 genes that were divided into two categories. (F) GO enrichment analysis results of overlapped genes. (J) The heatmap showed the expression pattern of the genes in ‘ion transport’ terms. The gene with red font was the putative target gene *HaHAK11*. The gene expression level was transformed with a *Z*-score value.

To identify downstream target genes of HaCBF4, we collected samples from *HaCBF4*-silenced plants and control plants under salt stress for transcriptome sequencing and data analysis (Fig. 4). After trimming low-quality reads and removing adaptor sequences, the clean data were aligned to the reference genome with an average 92.74% (92.19% ~ 93.23%) alignment rate. Unique mapped reads (average aligned rate 78.91%, 75.52% ~ 81.26%) were retained for subsequent gene quantification. After generating the gene expression matrix, we assessed the reproducibility of biological replicates by calculating the PCC and performing PCA using all gene expression levels (Fig. 4A and B), which showed high repeatability. A total of 7 726 differentially expressed genes (DEGs) were identified, comprising 3 633 upregulated and 4 093 downregulated genes (Fig. 4C). Furthermore, we screened for genes containing at least one DRE element within their 2 kb promoter regions. Comparative analysis identified 2 289 DEGs containing DRE elements, consisting of 1 095 upregulated and 1 194 downregulated genes (Fig. 4D, E and Table S9).

To explore the function of these potential target genes, we conducted GO enrichment analysis. We found that these genes were significantly enriched in terms such as ‘response to stimulus’ (*P* = 2.80E−17), ‘response to stress’ (*P* = 2.10E−14), ‘response to osmotic stress’ (*P* = 6.40E−06), ‘response to salt stress’ (*P* = 8.40E−06), ‘ion transport’ (*P* = 1.30E−03), and ‘transport’ (*P* = 6.10E−06) (Fig. 4F). A total of 74 genes were identified in the ‘ion transport’ category. Among them, we focused on the downregulated gene *LOC110908914* (hereafter referred to as *HaHAK11*), a member of the High-Affinity K^+^ transporter (HAK) family. Its rice homolog, *OsHAK11*, is known to be involved in root K^+^ uptake [37] (Fig. 4G). Therefore, we hypothesized that *HaHAK11* acts as a downstream target gene of HaCBF4.

### HaCBF4 directly binds to the DRE element in the promoter of *HaHAK11*

**Figure 5:**
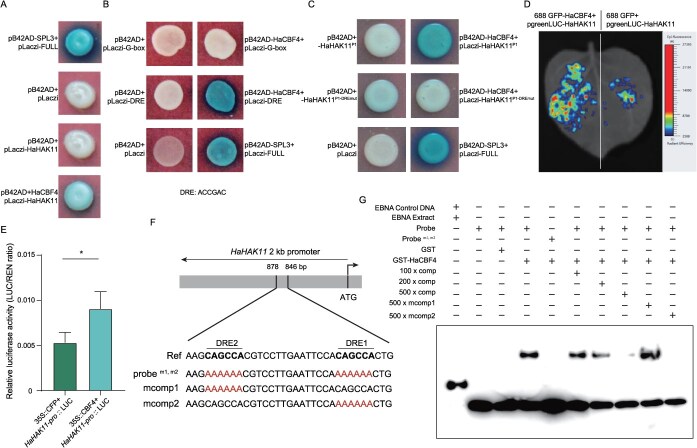
HaCBF4 directly binds to the DRE motif of the target gene *HaHAK11* promoter. (A, B) Y1H assay shows that HaCBF4 directly binds to the *HaHAK11* promoter (A) and the DER motif (B). (C) Y1H assay shows that HaCBF4 directly binds to the P1 fragment that contains two DER motifs. (D) Luciferase activity assay shows that co-expression of *HaCBF4* effectively activates the LUC reporter gene driven by the *HaHAK11* promoter. (E) Relative luciferase activity. Significance was determined using the paired Student’s *t*-test method. (* represents *P* < 0.05). (F) Schematic diagram of the probe. Putative HaCBF4 binding sites in the *HaHAK11* promoter regions. Two DRE motifs were located in the 846–878-bp region of the *HaHAK11* promoter, in which three probes (probe ^m1, m2^, mcomp1, mcomp2) were set using a mutated sequence at the DRE motif. (G) Electrophoretic mobility shift assays (EMSAs) of GST-HaCBF4 binding to the *HaHAK11* promoter. EBNA control DNA and EBNA extract (positive control) showed the expected band shift. Negative controls (free probe and GST + probe) exhibited no shift. Probes were labeled with biotin competition assays for the biotin-labeled probe competition assays were conducted by adding 50- to 500-fold unlabeled intact probe and 500-fold unlabeled mutant probes. Comp, the competitor probe; mComp, the mutant competitor probe.

To elucidate the mechanism by which HaCBF4 regulates the downstream target gene *HaHAK11*, we performed the Yeast one-hybrid (Y1H) assays. These assays confirmed that HaCBF4 binds to the promoter of *HaHAK11* (Fig. 5A). It has been reported that many DREB proteins in the AP2/ERF family bind to the DRE (A/GCCGAC) element, which is responsive to drought, cold, and ABA [22, 38]. Consistent with this, we confirmed that HaCBF4 specifically binds to the DRE element using the Y1H assay (Fig. 5B). Sequence analysis of the *HaHAK11* promoter revealed the presence of two DRE elements upstream of the transcriptional start site (TSS). Furthermore, the Y1H assay with HaHAK11^P1-DRE mutant^ (P1: fragment from 801 to 901 bp) demonstrated that HaCBF4 failed to bind when the DRE motifs were mutated (Fig. 5C).

Meanwhile, the promoter of *HaHAK11* was recombined into the vector pGreen-LUC to generate a reporter system, which was then transformed into tobacco leaves along with different effectors (HaCBF4 and empty GFP). This experiment showed that HaCBF4 could activate the activity of the *HaHAK11* promoter (Fig. 5D and E). Considering the two DRE elements (hereafter referred to as DRE1 and DRE2) in the *HaHAK11* promoter, we designed three probes named as probe ^m1, m2^ (mutated both elements), mcomp1 (mutated DRE2), and mcomp2 (mutated DRE1) to perform electrophoretic mobility shift assays (EMSAs). The results showed that HaCBF4 binds specifically to the second element (DRE2) of the *HaHAK11* promoter (Fig. 5F and G). Taken together, these results indicate that the HaCBF4 protein recognizes a specific element in the *HaHAK11* promoter and enhances transcriptional activation of this target gene.

### HaHAK11 regulates salt stress tolerance through the modulation of known salt-responsive gene expression levels

**Figure 6:**
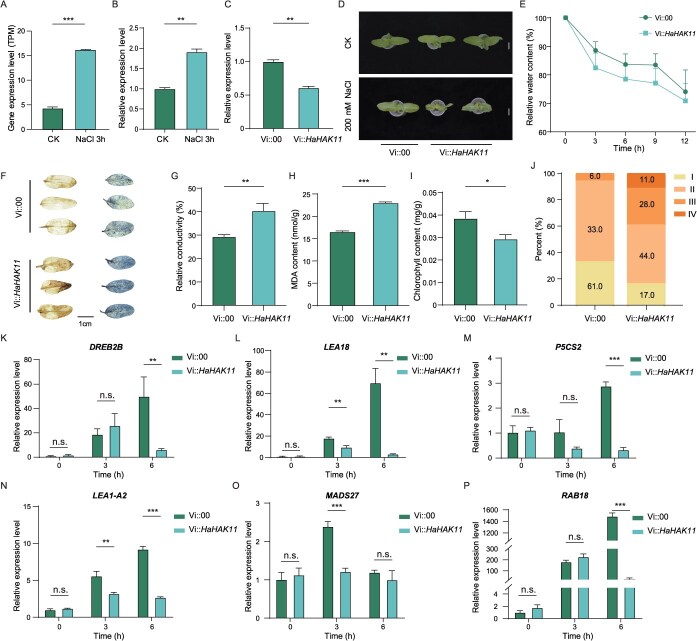
HaHAK11 positively regulates salt stress response by mediating the expression of known stress genes. (A) The gene expression level of *HaHAK11* under normal conditions and 3 h after 200 mM NaCl solution treatment at 3-week-old seedlings. (B) The relative gene expression level of *HaHAK11*. (C) The relative gene expression level of *HaHAK11* in gene silencing plants (Vi:: *HaHAK11*) was significantly decreased compared to the control plants (Vi:: 00). (D) The phenotype of Vi:: *HaHAK11* and Vi:: 00 after 5 days of treatment using a 200 mM NaCl solution at 3-week-old seedlings. (E) The relative WC of Vi:: *HaHAK11* and Vi:: 00 after treatment using a 200 mM NaCl solution. (F) The DAB and NBT content of Vi:: *HaHAK11* and Vi:: 00 leaf after NaCl treatment. (G–I) The relative conductivity (G), MDA content (H), and Chlorophy II content (I) of Vi:: *HaHAK11* and Vi:: 00 under salt stress conditions. (J) The proportion of four damaged degrees of leaf of Vi:: *HaHAK11* and Vi:: 00 after treatment. (K–P) The relative expression level of *DREB2B* (K), *LEA18* (L), *P5CS2* (M), *LEA1-A* (N), *MDAS27* (O), *RAB18* (P) of Vi:: *HaHAK11* and Vi:: 00 under normal and salt stress conditions, where time 0 represent samples without salt stress treatment, time 3 and 6 represent samples after 3- and 6-h salt stress treatment, respectively. Significance in A–C, G–I, and K–P was determined using the paired Student’s *t*-test method. ^*^, ^**^, and ^***^ represent *P* < 0.05, *P* < 0.01, and *P* < 0.001, respectively. n.s. means no significant difference.

As shown in Fig. 4, HaCBF4 directly binds to the *HaHAK11* promoter and enhances its transcriptional activation. Therefore, we wanted to investigate the role of HaHAK11 in sunflower salt stress response. First, we analyzed the expression pattern using a multi-tissue transcriptome and found that the gene was expressed in most tissues at different development stages (Fig. S5A and Table S8). Furthermore, the expression level of *HaHAK11* was induced by salt stress (Fig. 6A and B), which was consistent with the expression of *HaCBF4* (Fig. 3A and B). Sunflower transiently transformed for *HaHAK11* silencing using the VIGS method were constructed, and the transformation efficiency was confirmed using the qRT-PCR method (Fig. 6C). Gene-silenced sunflower plants for *HaHAK11* showed no significant difference compared with the control plants under normal conditions (Fig. S5B–F), but displayed a salt-sensitive phenotype compared to the controls under salt stress (Fig. 6D).

The relative WC of the *HaHAK11*-silenced plants showed a similar trend to the *HaCBF4-*silenced plants, decreasing more rapidly than the control group (Fig. 6E). The average intensity of DAB and NBT in *HaHAK11*-silenced plants was higher than the control under salt stress (Fig. 6F). The relative conductivity and MDA content of *HaHAK11*-silenced plants were significantly higher than those of the control group under salt stress (*P* < 0.01), while the Chlorophy II content was significantly lower (*P* < 0.05) (Fig. 6G–I). The proportion of damaged leaves in *HaHAK11*-silenced plants was higher than in the control group (Fig. 6J). These results indicate that *HaHAK11* also plays a positive role in sunflower salt stress response.

To understand how HaHAK11 regulates sunflower salt tolerance, we examined the expression levels of six known salt stress-responsive genes: *DREB2B* (*LOC110912968*), *LEA18* (*LOC110930290*), *P5CS2* (*LOC110865391*), *LEA1-A* (*LOC110917531*), *MADS27* (*LOC110883766*), and *RAB18* (*LOC118488043*) [39, 40] (Fig. 6K–P). The expression of these genes showed no significant difference between genotypes under normal conditions. However, while salt stress induced their expression in both control and *HaHAK11*-silenced plants, the level of induction was significantly reduced in the *HaHAK11*-silenced plants (Fig. 6K–P). This suggests that HaHAK11 mediates salt stress responses specifically by regulating the expression of these known salt-responsive genes. Thus, our results demonstrate that *HaHAK11* serves as a critical downstream target gene of HaCBF4 and plays an important role in salt stress responses.

## Discussion

Salt tolerance is a complex and important agronomic trait; hence, identifying genetic loci to reveal the molecular mechanism is a critical step for breeding ST crops. However, due to limited research on sunflowers, salt-responsive genes remain largely unidentified at the genome-wide scale. In this study, we performed GWAS using high-throughput phenotyping at the germination stage under salt stress conditions, and identified a total of 359 trait-associated SNPs and 63 InDels corresponding to 424 and 83 genes, respectively (Fig. 2). High-throughput phenotyping has been proven to be an effective tool for identifying genes when combined with GWAS analysis. This approach has been successfully used to reveal the genetic architecture of drought stress [8–11, 14], plant height [13], and root system [12]. In sunflowers, only a few studies have investigated the genetic architecture of abiotic and biotic stress via GWAS using traditionally measured traits, such as disease resistance [15, 16, 18] and drought stress [17], indicating that gene mining in sunflowers using GWAS is feasible. Here, we acquired 31 traits using high-throughput phenotyping under salt stress conditions, and performed GWAS to identify genetic determinants. This study provides an effective pipeline for salt stress-responsive gene identification that can be directly applied to other plant species.

The AP2/ERF family of TFs plays critical roles in the response to environmental stresses. Although the DREB subfamily proteins are known to directly bind to the DRE (CRT) element in the promoters of ABA, drought, and cold-responsive genes [21, 22, 35], their specific functions in salt tolerance remain unclear. Here, we demonstrate that the sunflower DREB protein HaCBF4 directly binds to the DRE motif in the promoter of the downstream gene *HaHAK11* and activates its expression, thereby establishing a HaCBF4-*HaHAK11* module in response to the salt stress (Figs S4–S6). In *Arabidopsis*, the *CBF1*/*2*/*3* are induced by cold stress, whereas *CBF4* is induced by drought stress [35]. Additionally, transgenic rice plants expressing the homolog *OsDREB1F* showed enhanced salt tolerance [41], although the molecular mechanism underlying the *OsDREB1F* response to salt stress has not been reported. Our findings indicate that *HaCBF4*- and *HaHAK11*-silenced plants exhibit increased salt sensitivity, which strongly supports their positive regulatory role in the salt stress responses. Furthermore, the expression level of the six salt-responsive genes (*DREB2B*, *LEA18*, *P5CS2*, *LEA1-A*, *MADS27*, and *RAB18*) in *HaHAK11*-silenced plants was significantly reduced under salt stress (Fig. 6K–P). This indicates that the HaCBF4-*HaHAK11* module regulates the expression of known salt-responsive genes (Fig. 6). Collectively, our results established a regulatory link between HaCBF4 and HaHAK11 and expanded our understanding of the function of AP2/ERF family proteins in salt stress response.

The Na^+^ preferential transporters encoded by HAK family genes modulate salt tolerance in crops by promoting Na^+^ exclusion from the shoot tissue [28–30]. In this study, we identified a HAK family gene, *HaHAK11,* as a key downstream target gene using integrative analysis (Fig. 5). The *HaHAK11*-silenced plants displayed a salt-sensitive phenotype compared to the control plants under salt stress conditions (Fig. 6C–J), consistent with the phenotype of the *Oshak12* mutant in rice and of *ZmHAK4*-knockout maize plants [28, 29]. We next focused on understanding the transcriptional changes following the loss of HaHAK11 function and found that the expression of several known salt stress-responsive genes was significantly reduced in the *HaHAK11*-silenced plants under salt stress (Fig. 6K–P). Since both OsHAK12 and ZmHAK4 function in shoot Na^+^ exclusion [28, 29], we hypothesized that HaHAK11 may also function in this physiological process. However, further studies are required to explore the specific mechanism. In this study, we primarily investigated the transcriptional regulation associated with HaHAK11, reflecting the downstream outcomes of salt stress response. Our experiments showed that *HaHAK11*-silenced plants exhibited higher ROS accumulation at the seedling stage under salt stress compared to control plants (Fig. 6F). Based on these findings, we propose that HaHAK11 may act as an initiator of signal transduction, regulating ion homeostasis and subsequently modulating the ROS signaling pathway, ultimately influencing gene expression during salt stress response. However, further research is needed to validate this model.

In summary, the combination of high-throughput phenotyping and GWAS identified genetic determinants of salt stress response in sunflower. We further showed that the candidate HaCBF4 directly binds to the DRE element in the promoter of its target gene *HaHAK11*, forming the HaCBF4-*HaHAK11* module that positively regulates salt tolerance in sunflower. These findings provide mechanistic insight and valuable genetic resources for molecular breeding aimed at improving salt stress tolerance in sunflower.

## Materials and methods

### Plant materials and treatment conditions

The sunflower oilseed variety AZB was selected as the background genotype for generating gene-silencing plants. All transgenic and control plants were grown in a phytotron in Hangzhou Normal University, Hangzhou city, Zhejiang Province, China (30°16′ N, 120°12′ E) under the following conditions: long-day photoperiod, 16 h light/8 h dark cycles, 150 μmol m^−2^ s^−1^ light intensity, and 25°C environment temperature. After germination for 2 days (d) on moist paper, seeds were transferred to containers containing 1/5-strength Hoagland nutrient solution, which was refreshed every 3 days. The 2-week-old seedlings were treated with 200 mM NaCl, and samples were collected at 0 h, 3 h, 6 h, 9 h, 12 h, and 5 days after treatment. Samples were immediately frozen in liquid nitrogen and stored at −80°C for subsequent experiments.

For phenotypic trait collection of 342 sunflower germplasm lines (Table S1) at the germination stage under salt stress, a dataset was provided in our collaborator’s previous study [8, 9]. Salt tolerance of sunflower germplasm lines was evaluated using membership function values (MFV). Based on the MFV, these germplasm lines were classified into three categories: salt tolerant or ST, moderately salt tolerant or MST, and salt sensitive or SS. For trait measurement, dehulled seeds were sown in Petri dishes and cultured in a phytotron in Xinjiang Academy of Agricultural Sciences, Urumqi City, China (43°49′21″N, 87°36′45″E). Eleven seeds of each germplasm line were sown and treated with either 300 mM NaCl solution or distilled water (control). Four biological replicates were set for each treatment. The number of germinated seeds was recorded daily for 7 days, and seedlings were collected on day 7 for trait measurements. In total, 31 traits, such as RL, FW, GE, GR, GI, GVI, and WC, were obtained. Detailed trait descriptions are provided in Table S2.

### Genome-wide association study

The GWAS was performed using GEMMA software (https://github.com/genetics-statistics/GEMMA) with the MLM that accounted for kinship and population structure. We then calculated the effective numbers of SNPs to determine the significance cutoff for the GWAS results using the GEC software with default parameters (https://pmglab.top/gec/). A total of 727 418 SNPs were selected, and the *P*-value cutoff by Bonferroni correction for family-wise error rate 0.05 is 6.87E−08. All 31 traits were used for GWAS analysis. Genome-wide LD decay was analyzed using the popLDdecay software, and the physical distance at which *r*^2^ decayed to 0.2 was used as the LD decay distance (~400 kb). Based on the significant associated SNPs and the LD decay distance, we merged consecutive significant SNPs if the distance between adjacent variants was less than 400 kb, extending the region until the distance to the next significant SNP/InDel exceeded the LD decay distance. Each merged region was defined as a GWAS locus. Genes containing at least one significant variant within their boundaries were considered potential candidate genes.

### Phylogenetic analysis

Amino acid sequences from *Arabidopsis*, rice, maize, peanut, and oilseed rape, etc. were obtained from the NCBI database. The sequences were aligned using the ClustalW algorithm, and phylogenetic relationships were inferred using the maximum likelihood method in MEGA-X software [42].

### Plasmid construction and plant transformation

To generate the *p35S: HaCBF4-GFP* construct, the coding sequence (CDS) of *HaCBF4* was amplified and recombined into the *p688-GFP* vector (digested with *XhoI*) for subcellular localization analysis and promoter activity validation.

To construct *pTRV2-CBF4* and *pTRV2-HaHAK11*, primers containing *BamHI* and *EcoRI* restriction sites were employed to amplify a 244-bp fragment from the *HaCBF4* CDS and a 264-bp fragment from the *HaHAK11* CDS. The amplified fragments were then ligated into the *pTRV2* vector (double-digested with *BamHI* and *EcoRI*), yielding *pTRV2-HaCBF4* and *pTRV2-HaHAK11*. These vectors were introduced into *Agrobacterium tumefaciens* strain GV3101 for virus-induced gene silencing of *HaCBF4* and *HaHAK11* in sunflower.

To generate *pB42AD-HaCBF4*, *pLaczi2μ-HaHAK11*, *pLaczi2μ-HaHAK11 P1*, *pLaczi2μ-HAK11 P1-DREmut*, and *pLaczi2μ-DRE*, the full-length *HaCBF4* CDS was amplified and recombined into the *pB42AD* vector (digested with *EorR1*). Additionally, the full-length promoter of *HaHAK11* was amplified and cloned into the *pLaczi2μ* vector to obtain *pLaczi2μ-HaHAK11*. A P1 upstream fragment (801–901 bp) of the *HaHAK11* promoter was also amplified and cloned into this same vector to generate *pLaczi2μ-HaHAK11 P1*. Furthermore, mutations were introduced into the DRE motif (ACCGAC) within the P1 segment of the *HaHAK11* promoter, generating *pLaczi2μ-HaHAK11P1-CRTmut*, and three tandem copies of the DRE motif fragments were cloned into *pLaczi2μ* to generate *pLaczi2μ-DRE*. These constructs were used for Y1H assays.

To construct *pGreen-HaHAK11*, the promoter region of *HaHAK11* was amplified and recombined into the *pGreen-0800-LUC* vector (digested with *NcoI*) for a luciferase-based assay to validate promoter activity.

Finally, to generate *GST-HaCBF4*, the full-length *HaCBF4* CDS was amplified and cloned into the *pGEX-4T-1* vector (double digested with *BamH I* and *EcoR I*) for recombinant HaCBF4 protein expression. All primers used for vector construction are listed in Table S10.

### Subcellular localization

The full-length coding sequence of *HaCBF4* was cloned into the p688-GFP vector (XhoI) in-frame and upstream of the GFP sequence, and transiently expressed in leaves of *N. benthamiana* as described previously [43].

### Virus-induced gene silencing

For the VIGS experiments, the coding DNA sequence of *HaCBF4* (88–313 bp) and *HaHAK11* (1846–2114 bp) was amplified and recombined into the binary pTRV2 vector, respectively. pTRV1, pTRV2, pTRV2-*HaCBF4*, and pTRV2-*HaHAK11* were introduced into *Agrobacterium tumefaciens* strain GV3101 and stored in 30% glycerol at −80°C. For initial activation, 200 μl of the bacterial stock was inoculated into 2 ml of LB medium. Following activation, the culture was transferred to 100 ml of fresh LB medium and incubated with shaking until the OD600 reached 0.8–1.0 (28°C, 220 rpm). Bacterial cells were harvested by centrifugation and resuspended in infection buffer (10 mM MES, 10 mM MgCl₂, 200 μM acetosyringone [AS]). The Agrobacterium cultures carrying the gene constructs were mixed at a 1:1 ratio with the pTRV1- containing bacterial suspensions. AZB seeds were surface-sterilized, soaked in sterile water for 1–2 days, manually de-coated, scratched, and immersed in the prepared bacterial suspension. The inoculated seeds were incubated in the dark at 28°C for 6 h. Subsequently, the seed-containing suspension was vacuum-infiltrated three times using a vacuum pump, with each cycle lasting 5 min. After infection, the seeds were sown on sterile filter paper for germination. Gene silencing efficiency was confirmed by RT-PCR analysis.

### RNA extraction, library preparation, sequencing, and data analysis

Total RNA was extracted from the samples using the RNeasy Plus Mini Kit (Vazyme), and cDNA libraries were prepared with NEBNext Ultra RNA Library Prep Kit (NEB). After quality inspection, high-quality libraries were sequenced on the Illumina HiSeq X-ten sequencing platform (Illumina). The transcriptome analysis pipeline was performed as previously described [44]. After trimming low-quality reads (*Q* < 20) and adapter sequences, clean data were aligned to the reference genome (HanSK) using HISAT2 software with default parameters [45]. Gene expression levels were quantified using featureCounts with default parameters [46]. PCCs between biological replicates were calculated to evaluate reproducibility based on gene counts. DEGs were identified using the cutoff of |fold-change| ≥ 1.2 and *P* < 0.05. Read counts were also converted to fragments per kilobase of exon model per million mapped fragments (FPKM) value to display gene expression level. GO enrichment analysis of DEGs was performed with the agriGO v2 webtool (https://systemsbiology.cau.edu.cn/agriGOv2), and these terms with *P* < 0.05 were determined to be significantly enriched.

### Measurement of water loss rate and ROS content

Samples collected at 0, 3, 6, 9, and 12 h after salt stress treatment from gene-silenced and control plants were used to measure FW. The water loss rate at each time point was calculated as the corresponding FW divided by the 0 h FW. Samples collected after 6 h of salt stress treatment were used to measure ROS content. Leaves were incubated overnight in the dark at room temperature in NBT (1.0 mg·ml^−1^ NBT dissolved in 25 mM HEPES buffer (pH = 7.6)) and DAB (1.0 mg·ml^−1^ DAB dissolved in 50 mM NaAC buffer (pH = 3.8)), then washed with 95% ethanol until the chlorophyll was completely removed.

### Electrophoretic mobility shift assay

Strain BL21 (DE3) harboring the GST-HaCBF4 plasmid was cultured until the OD_600_ reached approximately 0.6. Protein expression was induced by adding 0.2 mM IPTG, followed by overnight incubation at 25°C. Bacterial cells were collected by centrifugation, resuspended in PBS buffer supplemented with 1 mM PMSF, and lysed by ultrasonication. The cell lysate was then centrifuged to obtain the supernatant. The supernatant was incubated with glutathione agarose beads at 4°C for 3 h, after which the beads were washed three times with PBS buffer. Bound GST-HaCBF4 protein was eluted using 10 mM reduced glutathione in 50 mM Tris–HCl (pH = 8.0). Protein purity was assessed by SDS-PAGE, and the purified protein was stored at −80°C.

For electrophoretic mobility shift assays (EMSAs), the GST-HaCBF4 protein was incubated with biotin-labeled probes, mutant probes, or unlabeled competitor probes at room temperature for 20 min using a chemiluminescent EMSA kit. Electrophoresis was performed in 0.5× TBE buffer at 10 V/cm. Semi-dry transfer was carried out using 0.5× TBE buffer at 380 mA for 60 min. Binding signals were detected using a Streptavidin-HRP Conjugate working solution. The DNA sequences of the probes are provided in Table S10.

### Promoter-luciferase assay

The plasmids (p688-*GFP* and pGreen-*HaHAK11*) were transformed into the Agrobacterium GV3101 strain. The bacterial cultures were resuspended in infiltration buffer (10 mM MES,10 mM MgCl_2_, and 0.2 mM AS) and infiltrated into the leaves of *N. benthamiana*. The infiltrated leaves were subjected to 12 h of dark treatment, followed by 48 h of light. The treated leaves were then sprayed with 1 mM D-luciferin solution and allowed to remain undisturbed for 10 minutes. The luciferase activity was imaged using a CCD system.

### Quantitative real-time-PCR assay

High-quality RNA was reverse-transcribed using the HiScript II 1st Strand cDNA Synthesis Kit (+ gDNA wiper) (AG). Relative expression levels of target genes were measured on the SLAN-96S detection system using SYBR Green solution (Takara) and then were calculated using the 2^-△△CT^ method with the *HaTublin* gene as the internal control. Experiments were performed with three biological replicates, and the primers were listed in Table S10.

### Statistical analysis and reproducibility

Data are presented as the mean ± S.D., and the exact sample size used for statistical calculations is provided in each figure legend. All statistical analysis was performed using R software (https://www.r-project.org/). Statistical significance was determined using a two-sided Student’s *t*-test method, and the results with a *p*-value <0.05 were considered statistically significant (‘^*^,’ ‘^**^,’ ‘^***^,’ and ‘n.s.’ in figure legend represent *P* < 0.05, *P* < 0.01, *P* < 0.001, and no significant difference, respectively).

## Supplementary Material

Web_Material_uhag081

## Data Availability

All data are incorporated into the article and its online supplementary material. The raw data of transcriptome have been deposited in the Genome Sequence Archive (GSA) in the National Genomics Data Center (NGDC) with accession CRA036423. All data analysis code in this study has been uploaded to a GitHub repository (https://github.com/GUOWEIJUN/Sunflower_phenomics_analysis).
