# Supplementary material for: Integrating high-throughput phenomics and GWAS unravels the HaCBF4-HaHAK11 module to regulate salt stress tolerance in sunflower (Helianthus annuus L.)
Source: Hortic Res. 2026 Mar 4;13(7):uhag081. doi: 10.1093/hr/uhag081 (PMC13267642; doi:10.1093/hr/uhag081)
Supplement: Web_Material_uhag081 [file web_material_uhag081.zip › Supplementary Figure S1-S5-R2-clean.docx]

**Integrating high-throughput phenomics and GWAS unravels the HaCBF4-*HAK11* module to regulate salt stress tolerance in sunflower**

Guo *et al*.


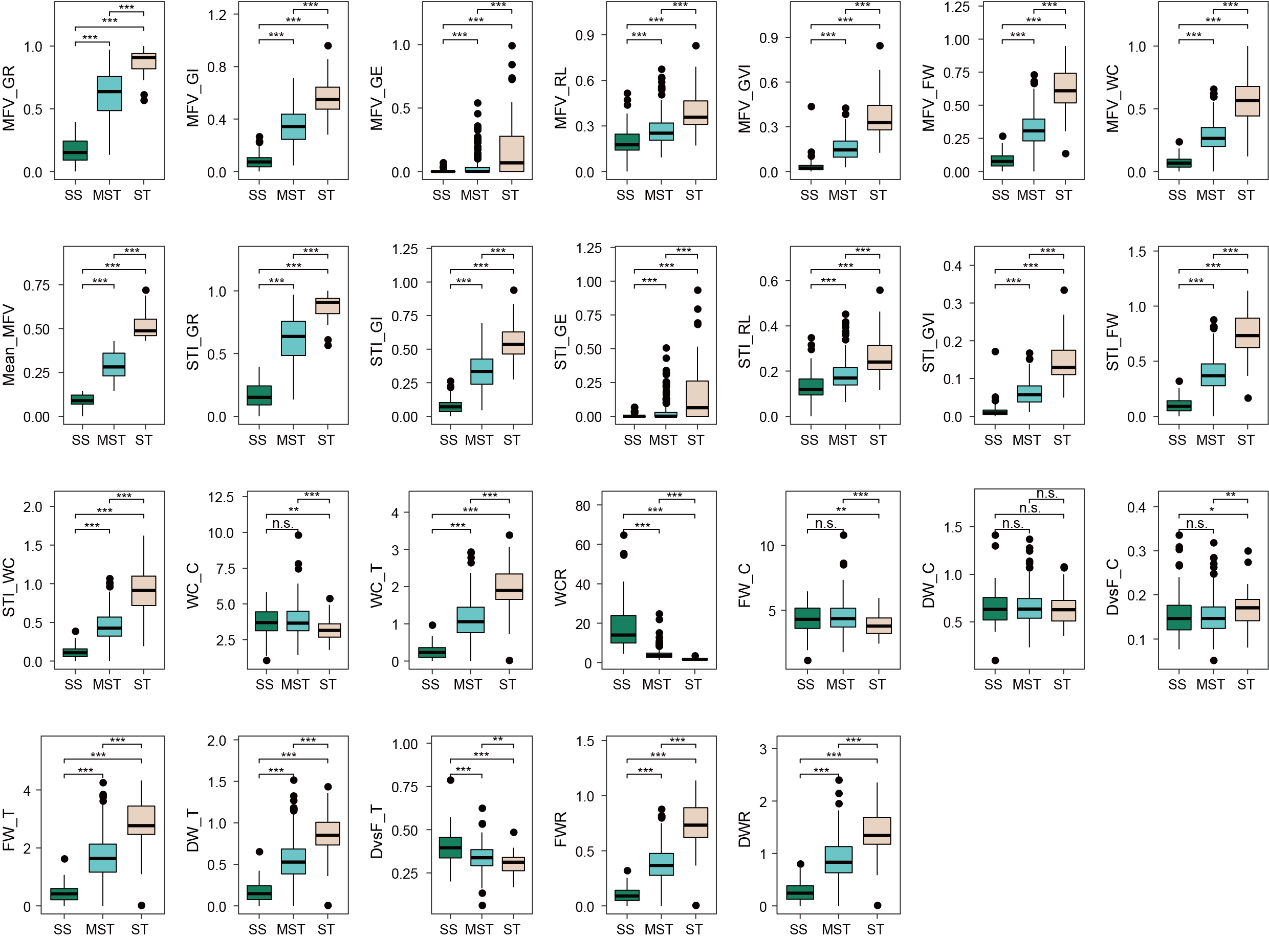
**Figure S1. Comparison of traits in three subpopulations with different salt tolerance.**

Comparison of 26 traits from high-throughput platform of 342 germplasms at germination stage under salt stress conditions. SS, MST, and ST represent salt sensitive, moderate salt tolerance, and salt tolerance, respectively. Significance was determined using paired Student’s *t*-test method. “*”, “**”, “**”, and n.s. mean p < 0.05, 0.01, 0.001, and no significant difference. MFV and STI represent Membership function values (MFV) and Salt tolerance coefficients (STI), respectively. GR, GI, RL, GVI, WC_C, WC_T, WCR, FW_C, DW_C, DvsF_C, FW_T, DW_T, DvsF_T, FWR, DWR represent Germination rate (GR), Germination index (GI), Root length (RL), Germination vigor index (GVI), Water content under normal condition (WC_C), Water content under salt stress (WC_T), Ratio of water content (treat/normal) (WCR), Fresh weight under normal condition (FW_C), Dry weight under normal condition (DW_C), Ratio of dry weight to fresh weight under normal condition (DvsF_C), Fresh weight of the seedlings under salt stress (FW_T), Dry weight of the seedlings under salt stress (DW_T), Ratio of dry weight to fresh weight under salt stress condition (DvsF_T), Ratio of fresh weight (treat/normal) (FWR), and Ratio of dry weight (treat/normal) (DWR), respectively. The definition of these traits was described in the Supplementary Table S2.


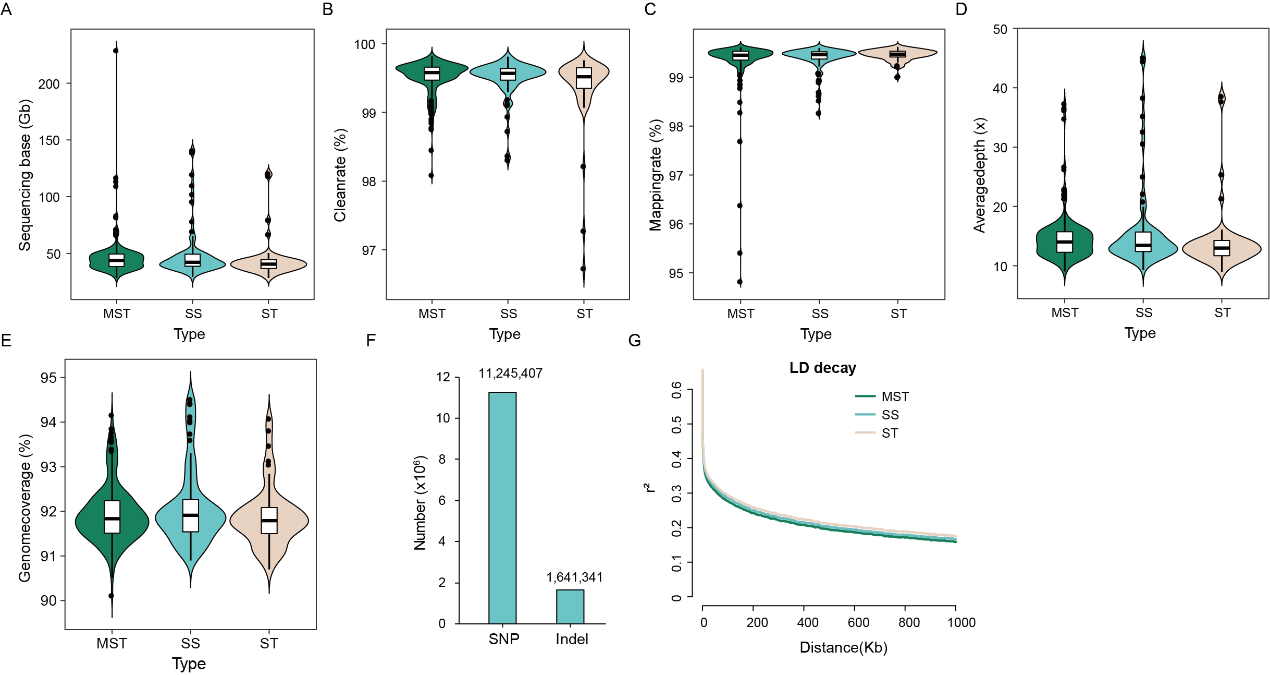


**Figure S2. The data analysis results of whole-genome sequencing in 342 germplasms.**

(**A-E**) Sequencing base (**A**), clean rate (**B**), mapping rate (**C**), average depth (**D**) and genome coverage (**E**) of 342 germplasms that were divided into three groups: 51 salt tolerance (ST), 218 moderate salt tolerance (MST), and 73 salt sensitive (SS). (**F**) Number of variations. (**G**) Genome wide average LD distance decayed to *r*^2^ = 0.2 for different salt tolerance lines.


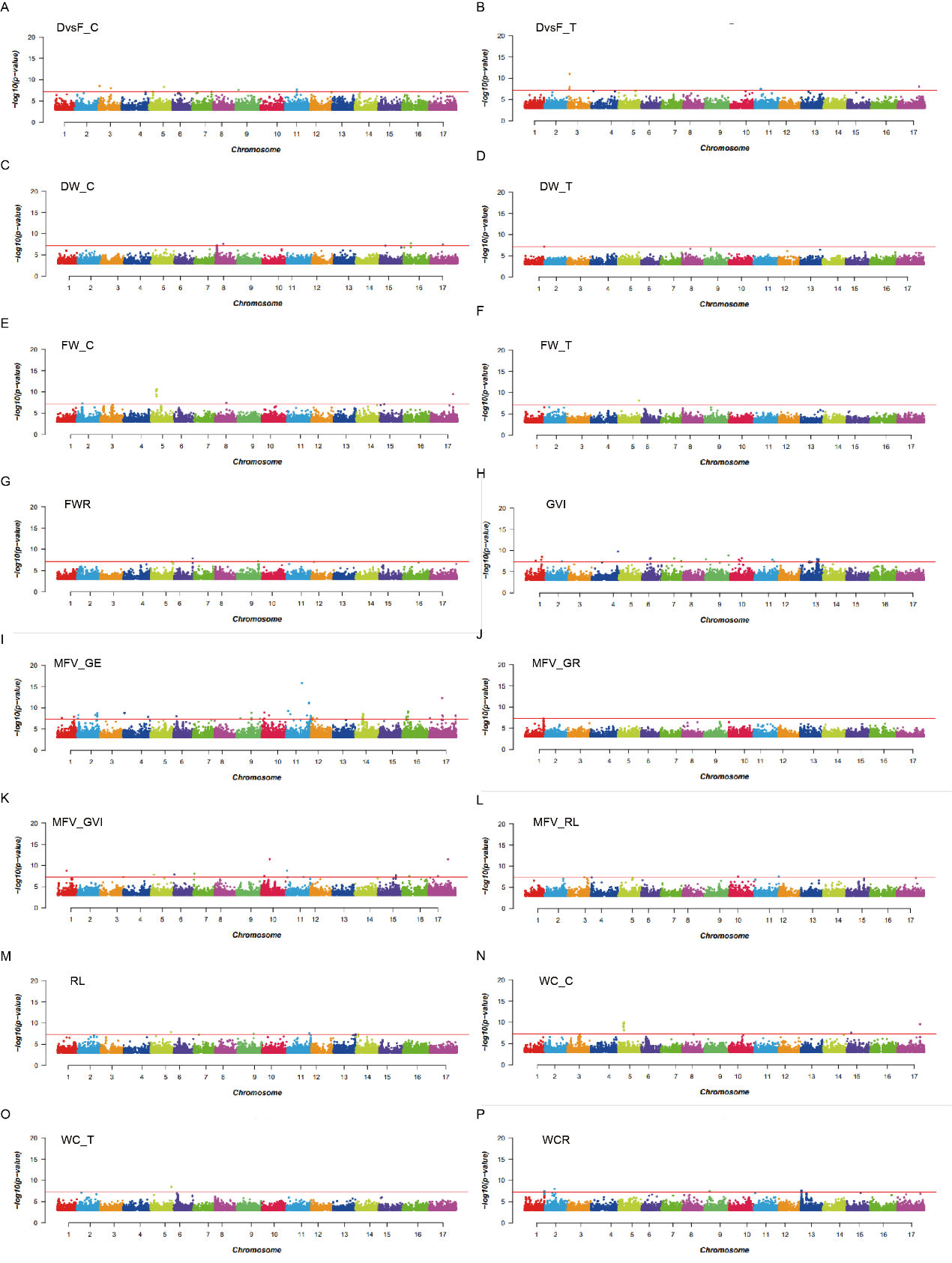
**Figure S3. Manhattan plot of traits with significantly associated signals.**

(**A-P**) The Manhattan plot of DvsF_C (**A**), DvsF_T (**B**), DW_C (**C**), DW_T (**D**), FW_C (**E**), FW_T (**F**), FWR (**G**), GVI (**H**), MFV_GE (**I**), MFV_GR (**J**), MFV_GVI (**K**), MFV_RL (**L**), RL (**M**), WC_C (**N**), WCR (**O**), WC_T (**P**). A detailed description of these traits was listed in Supplementary Table S2.


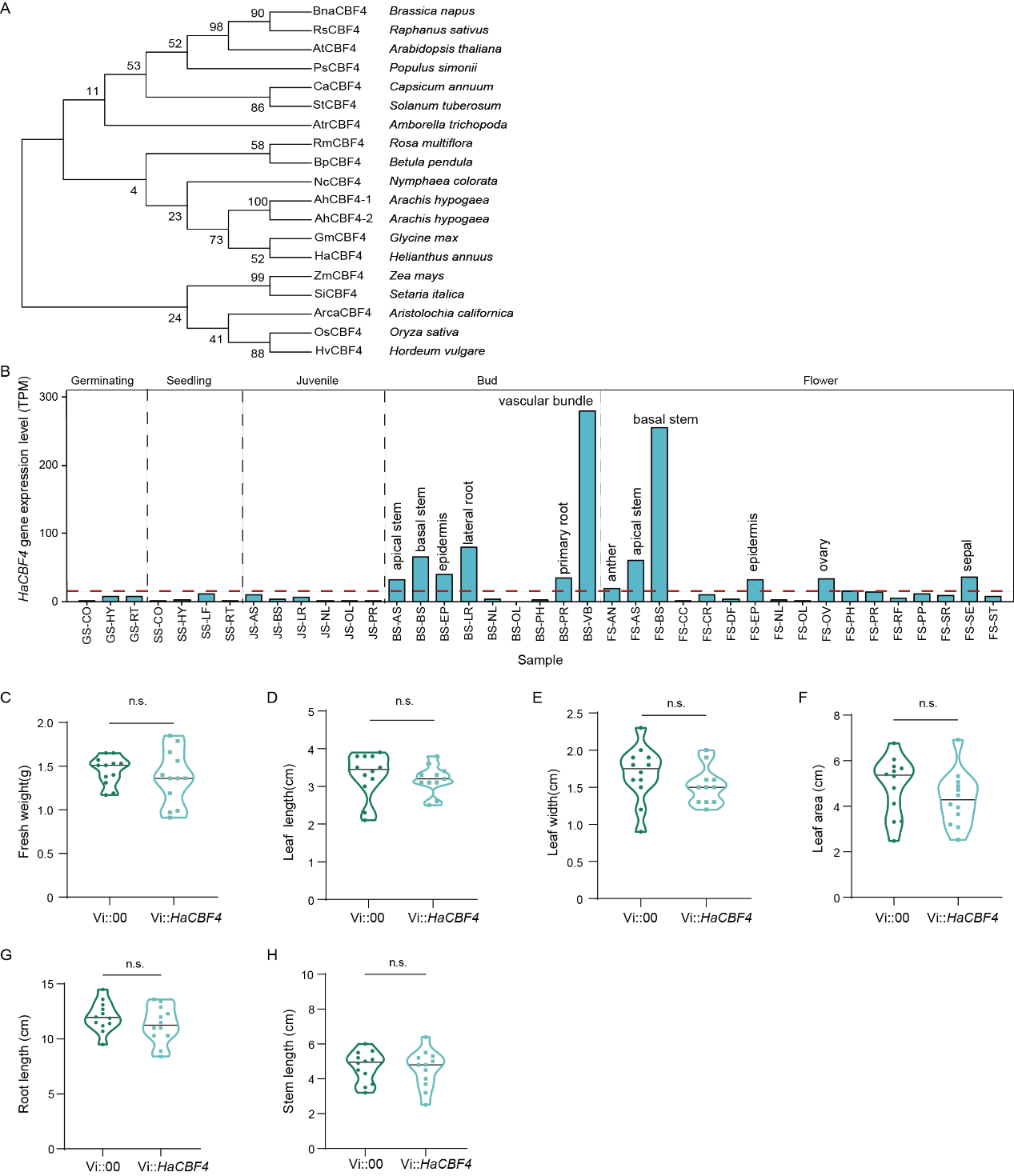
**Figure S4. Gene silencing of *HaCBF4* shows no significant change in the phenotype under normal conditions.**

(**A**) Phylogenetic tree of the HaCBF4. (**B**) The gene expression level of *HaCBF4* in different samples at different stages. GS, SS, JS, BS, and FS represent germinating, seedling, juvenile, bud, and flower stages, respectively. And the OL, PR, EP, PH, VB, AN, CC, CR, DF, OV, RF, PP, SR, SE, ST represent cotyledon, hypocotyl, root, leaf, apical stem, basal stem, lateral root, new leaf, old leaf, primary root, epidermis, phyllary, vascular bundle, anther, column cap, corolla, disk flower, ovary, ray flower, receptacle, secondary root, sepal, style sample, respectively. A detailed description was provided in Supplementary Table S8. (**C-H**) The fresh weight (**C**), leaf length (**D**), leaf width (**E**), leaf area (**F**), root length (**G**), and stem length (**H**) showed no significantly different change in gene silencing plants (Vi::*CBF4*) than the control plants (Vi::00). N = 12. Significance was determined using the paired Student’s *t*-test method. n.s. means no significant difference.


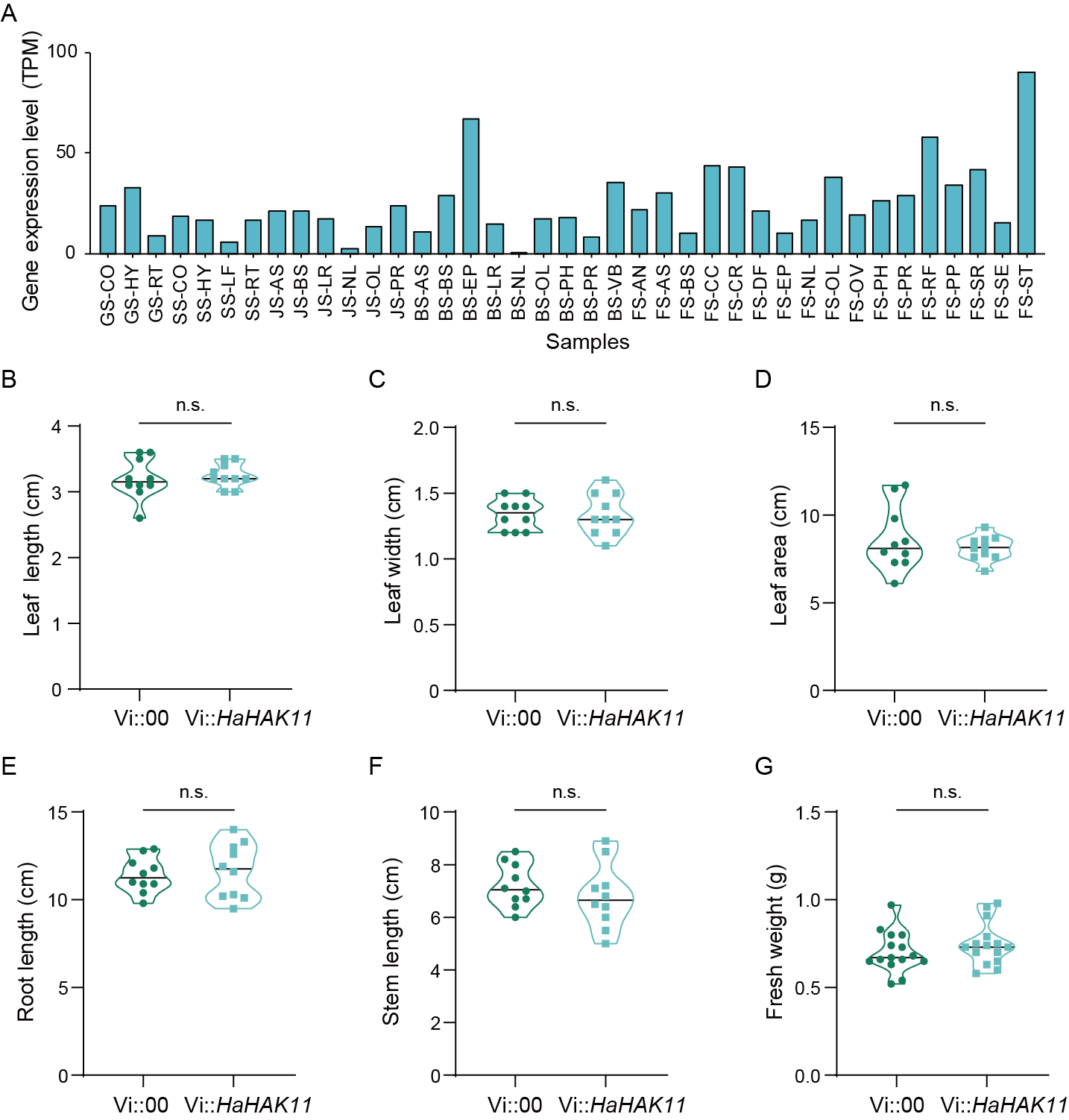
**Figure S5. Gene silencing of *HaHAK11* shows no significant change in the phenotype under normal conditions.**

(**A**) The gene expression level of *HaAKT11* in different samples at different stages. A detailed description of the sample name was provided in Supplementary Table S8. (**B-F**) The leaf length (**B**), leaf width (**C**), leaf area (**D**), root length (**E**), stem length (**F**), and fresh weight (**G**) showed no significantly different change in gene silencing plants (Vi::*HaHAK11*) than the control plants (Vi::00). N = 10. Significance was determined using the paired Student’s *t*-test method. n.s. means no significant difference.
